# Decline in physical activity during adolescence is not associated with changes in mental health

**DOI:** 10.1186/s12889-016-2983-3

**Published:** 2016-04-07

**Authors:** Martin L. Van Dijk, Hans H. C. M. Savelberg, Peter Verboon, Paul A. Kirschner, Renate H. M. De Groot

**Affiliations:** Welten Institute, Faculty of Psychology and Educational Sciences, Open University of the Netherlands, Heerlen, Netherlands; Fontys Sporthogeschool, School of Sport Studies, Fontys University of Applied Sciences, Eindhoven, The Netherlands; Department of Human Movement Sciences, NUTRIM (School for Nutrition and Translational Metabolic Research), Maastricht University, Maastricht, The Netherlands; Faculty of Psychology and Educational Sciences, Open University of the Netherlands, Heerlen, Netherlands; Department of Epidemiology & Complex Genetics, NUTRIM (School for Nutrition and Translational Metabolic Research), Faculty of Health, Medicine and Life Sciences, Maastricht University, Maastricht, The Netherlands

**Keywords:** The GOALS study, Exercise, Accelerometry, Depressive symptoms, Self-esteem, Youth

## Abstract

**Background:**

The majority of studies investigating associations between physical activity and mental health in adolescents have been cross-sectional in design. Potential associations between physical activity and mental health may be better examined longitudinally as physical activity levels tend to decrease in adolescence. Few studies have investigated these associations longitudinally in adolescents and none by measuring physical activity objectively.

**Methods:**

A total of 158 Dutch adolescents (mean age 13.6 years, 38.6 % boys, grades 7 and 9 at baseline) participated in this longitudinal study. Physical activity, depressive symptoms and self-esteem were measured at baseline and at the 1-year follow-up. Physical activity was objectively measured with an ActivPAL3™ accelerometer during one full week. Depressive symptoms were measured with the Center for Epidemiologic Studies Depression Scale (CES-D) and self-esteem was assessed with the Rosenberg Self-Esteem Scale (RSE). Results were analysed using structural equation modelling.

**Results:**

Physical activity levels decreased 15.3 % over a 1-year period (*p* < .001), with significantly (*p* = .001) greater decreases during grade 7 (-20.7 %) than during grade 9 (-5.0 %). Overall, depressive symptoms decreased (-12.1 %, *p* < .001) over a 1-year period, while self-esteem did not change significantly (+2.9 %, *p =* .066). Higher levels of depressive symptoms at baseline predicted a greater decline in depressive symptoms (β = -.51, *p* < .001) and higher levels of self-esteem at baseline predicted a smaller increase in self-esteem (β = -.48, *p* < .001). The decline in physical activity did not appear to predict any change in depressive symptoms and self-esteem.

**Conclusion:**

The decline in physical activity over a 1-year period during adolescence is not associated with a change in mental health. Further studies in adolescents aiming to investigate whether a change in physical activity is associated with a change in mental health should control for baseline levels of mental health and academic year differences.

## Background

According to a recent review, 20 % of adolescents have mental health problems [1]. Left untreated, these adolescents have an increased risk of isolation, school absenteeism, academic underachievement, substance abuse, and suicide [2–4]. Physical activity has been shown to have beneficial effects on mental health in adolescents [5], particularly on depression (i.e. fewer depressive symptoms) and self-esteem [6]. Physical activity may alleviate depressive symptoms due to an increased release of β-endorphins, which are related to a positive mood and an overall enhanced sense of well-being [7]. Also, physical activity may directly increase self-esteem [8], or it may indirectly increase it by creating the perception of physical competence and an improved level of fitness [9, 10]. A number of studies on the relationship between physical activity and mental health in adolescents have reported positive associations (see reviews [11–13]); however, the research designs of these studies are often [6]. Most of the studies use cross-sectional designs [14]; however, physical activity levels tend to decrease during the adolescent period [15, 16], and longitudinal studies are therefore needed to understand the association between the decline in physical activity during adolescence and the change in mental health status. One limitation of the longitudinal studies investigating associations between changes in physical activity and changes in mental health in adolescents is that physical activity was measured subjectively through the use of questionnaires. This self-report method has several limitations [17], such as overestimating time spent in physical activities, social desirability [18] and recall bias [19]. Furthermore, self-reported physical activity may result in selective bias (i.e. non-systematic bias). For example, research showed that males, and those specifically with lower body mass index, are more likely to overestimate their physical activity levels [20]. In addition, there is some evidence for differential expression of social desirability by ethnicity [21] which may impact research findings. Therefore, it is important to use an objective instrument when investigating associations between changes in physical activity and changes in mental health.

Only three studies have investigated the association between changes in physical activity and changes in depressive symptoms in adolescents. Motl, Birnbaum, Kubik and Dishman [22] revealed that an increase in physical activity between the beginning of grade 7 and the end of grade 8 was inversely related to a change in depressive symptoms (i.e. fewer depressive symptoms). Also, Neissaar and Raudsepp [23] found in adolescent girls 11–12 years old that a decrease in physical activity across 2 years was inversely associated with a change in depressive symptoms (i.e. more depressive symptoms). In contrast, Rothon et al. [14] found no significant association between a change in physical activity and a change in depressive symptoms in adolescents progressing from grade 7 to grade 9. Also, three studies of adolescents reported associations between baseline levels of physical activity and changes in depressive symptoms between baseline and follow-up. Wiles et al. [24] reported that adolescents 11–14 years old who participated in at least 1-h of sports activity (e.g. football, tennis, swimming) on a daily basis had fewer emotional problems 1-year later. In addition, Stavrakakis, de Jonge, Ormel, and Oldehinkel [25] reported that high baseline levels of physical activity in adolescents 11–14 years old predict a decrease in depressive symptoms, such as depressed mood, low self-worth and loss of pleasure, 2 years later. In contrast, Clark et al. [26] found that physical activity levels at baseline were not significantly associated with depressive symptoms 2 years later in adolescents 11–14 years old.

Only one study of adolescents investigated the association between change in physical activity and change in self-esteem. Lindwall et al. [27] found no significant association in 14- and 15-year-old girls between change in physical activity and change in self-esteem over a 3-year follow-up period. Other studies in the field of physical activity and self-esteem in adolescents were experimental or cross-sectional in design. Boyd and Hrycaiko [28] examined the effect of a 6-week physical activity intervention on self-esteem in 9 to 16 years old adolescent girls. The results indicated that 9-and-10-year-old girls in grades 4 and 5 with low self-esteem benefitted from the intervention while no benefit was observed in the older girls. In addition, Tremblay, Inman, and Willms [29] found that regular participation in physical activities (e.g. sports, active commuting, stretching exercises and strength exercises) was positively associated with self-esteem in adolescents in grade 6. Finally, Schmalz, Deane, Birch, and Krahnstoever Davison [30] found, in 9- and 11-year-old girls, that higher levels of physical activity at baseline predicted higher levels of self-esteem 2 years later.

The main goal of the current study of adolescents in grades 7 and 9 was to investigate over 1 year the associations between changes in objectively measured physical activity and changes in depressive symptoms and self-esteem. It was hypothesized that a change in physical activity is positively associated with a change in mental health (i.e. fewer depressive symptoms and higher levels of self-esteem) because the majority of such studies on adolescents reported positive associations between physical activity and mental health [6, 11–13].

## Methods

### Study design and participants

The GOALS (Grootschalig Onderzoek naar Activiteiten van Limburgse Scholieren [Large-scale Research of Activities in Dutch Students]) Study was originally designed to investigate the associations between physical activity and cognitive performance and academic achievement. This study was conducted at a secondary school in the Netherlands. All students (*n* = 526) in grades 7 and 9 of senior general secondary education (SGSE) or university preparatory education (UPE) were invited to participate in GOALS and 440 students (83.7 %) of them participated. Participants with both complete baseline and follow-up data (*n* = 158) were included in the current study.

### Procedures

The Ethical Committee of the Open University of the Netherlands approved the research protocol (reference number: U2013/07405/HVM). Before starting data collection, information about the study including goals and procedures was distributed to students of the selected classes. Also, parents and/or guardians were invited to a presentation about the study. Parents and/or guardians were allowed to sign an objection form if they did not agree with the participation of their children, and the students themselves were given the same options. No written consent was obtained as the parents/guardians were informed of the study.

Data collection was executed by research assistants trained in standard protocols who were given standardised instructions. Physical activity levels of the participants were measured during a normal school week by wearing an accelerometer continuously for one full week, 24 h per day. Cardiovascular fitness was measured by the shuttle-run test. Experienced feelings of depressive symptoms and self-esteem were self-reported using validated questionnaires. After finishing the study and after full participation, participants received a gift voucher of 15 euro.

Using the same protocols, all baseline procedures were also executed at the 1-year follow-up. Data collection was conducted between October 2011 and May 2013 at a secondary school in the south of the Netherlands. A comprehensive description of the procedures of this study at baseline has been published elsewhere [31].

### Instruments

#### Physical activity

Accelerometers (model ActivPAL3™; Paltechnologies, Glasgow, UK) were used to measure physical activity. These accelerometers were taped onto the midpoint of the anterior part of the right thigh of the participants using Tegaderm™ (3 M, St. Paul, MN, US) transparent film. Data were processed with ActivPAL™ Professional software (version 6.4.1).

Physical activity was estimated using a maximum of six valid days (i.e., a day that the student wore the accelerometer for all 24 h) per student. According to prescribed accelerometer testing protocols, at least four valid days including both weekend days were required to measure physical activity [32]. The total physical activity volume per week was calculated by the total number of accelerometer steps per week. The change of physical activity over a 1-year period (∆PA) was calculated by the difference between the baseline and follow-up scores.

#### Depressive symptoms

Feelings of depressive symptoms were measured with the Center for Epidemiologic Studies Depression Scale (CES-D) designed by Radloff [33]. Using this 20-item self-report scale, participants rated the frequency of 20 depressive symptoms over the previous week. The answer options were the following: (0) Rarely or none of the time (on average less than 1 day), (1) Some or a little of the time (1–2 days), (2) Occasionally or a moderate amount of time (3–4 days), and (3) Most or all of the time (5–7 days). The positively worded items were reverse-scored. A total severity score was calculated by summing all items, ranging from 0 (not depressed) to 60 (a high number of depressive symptoms). The change in the number of depressive symptoms over the 1-year period was calculated by the difference between the baseline and follow-up CES-D scores (∆DS).

#### Self-esteem

The Rosenberg Self-Esteem Scale (RSE) was used to measure self-esteem [34]. The scale consists of ten statements dealing with general feelings about the participant’s own self-image. The statements were answered by: (0) Strongly agree, (1) Agree, (2) Disagree, (3) Strongly Disagree. The negatively worded items were reverse-scored. The self-esteem score was calculated by summing all items and ranges from 0 (minimum score of self-esteem) to 30 (maximum score of self-esteem). The change of self-esteem over a 1-year period was calculated by the difference between the baseline and follow-up RSE scores (∆SE).

#### Covariates

Sex was coded as -0.5 = boys and 0.5 = girls. Nationality was coded as -0.5 = native Dutch (i.e. both parents/guardians born in the Netherlands) and 0.5 = non-native Dutch. Academic year was coded as -0.5 = 7^th^ graders (i.e. the first year of secondary school in the Netherlands) and 0.5 = 9^th^ graders. Socioeconomic status was measured by the highest educational level of the parents/guardians. If the parents/guardians had at most a secondary vocational education level, socioeconomic status was coded as ‘low-medium’ = -0.5. In all other cases socioeconomic status was coded as ‘high’ = 0.5.

Body mass index (BMI) and cardiovascular fitness were objectively measured and included in the analyses as baseline covariates (i.e. baseline levels) and change covariates (i.e. difference in measurement between baseline and follow-up). Weight in kg (rounded up) and height in meters (two decimals) were measured in light clothing without shoes. The BMI was then calculated by dividing the weight by the height squared. The change of BMI over the 1-year period was calculated by the difference in BMI between the baseline and follow-up measurements. Maximum oxygen consumption (VO_2_ max) was used as indication of cardiovascular fitness and estimated by the 20-m shuttle run test [35]. A comprehensive description of the 20-m shuttle run test and calculation of the VO_2_ max has been published elsewhere [31]. The change of cardiovascular fitness over the 1-year period was calculated by the difference between the baseline and follow-up scores on the 20-m shuttle run test.

### Data analysis

The Statistical Package for Social Sciences (SPSS) for Windows (version 19.0; SPSS, Inc, Chicago, Illinois) was used for paired sample *t* tests and independent sample *t* tests. Structural equation modelling was performed in R [36] with the package Lavaan [37]. The distributions of the independent and dependent variables at both baseline and follow-up were normal. The level of significance was .05 in all analyses.

Associations between changes of physical activity over time and changes in depressive symptoms and self-esteem were analysed by structural equation modelling (SEM). SEM was used to simultaneously estimate various associations in the data. Not only the effects on the dependent variables were modelled, but also other relevant correlations between independent variables and covariates were incorporated into the model. The model consisted of one independent variable (∆PA) and two dependent variables (∆DS and ∆SE). Relationships were controlled for physical activity at baseline (PA_Baseline), depressive symptoms at baseline (DS_Baseline) and self-esteem at baseline (SE_Baseline), as well for covariates of sex, nationality, academic year, socioeconomic status, BMI and cardiovascular fitness.

Only complete cases (*N* = 158) were used in the analyses. Missing data were not imputed for two reasons. We did not have a valid predictive model to be used for imputation. A naive or improper imputation method could have given biased results. Second, additional analyses of the baseline data showed no significant differences on the variables of interest between the final sample and the drop outs (for more information see Results section).

Analyses were also conducted using moderate-to-vigorous intensity physical activity as an independent variable. Moderate-to-vigorous intensity physical activity was calculated by the total number of accelerometer steps with a cadence ≥ 100 steps/min in accordance with many studies [38]. These results showed no relevant differences compared to analyses using the total physical activity volume per week as an independent variable and therefore have not been published.

## Results

**Fig. 1:**
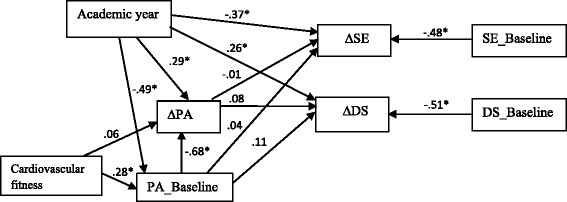
Schematic overview of structural equation modelling. Change in objectively measured physical activity (∆PA) associated with change in self-esteem (∆SE) and depressive symptoms (∆DS). Only most important paths are presented for readability. SE_Baseline = baseline levels of self-esteem. DS_Baseline = baseline levels of depressive symptoms. PA_Baseline = baseline levels of physical activity measured objectively by accelerometer. * indicates statistical significance at *p* < .05

The descriptive statistics are shown in Table 1. The structural equation model (Fig. 1), built to test whether a decline in physical activity was related to a change in mental health, fitted the data well (χ^2^ = 23.5, *df* = 12; CFI = .97; TLI = .92; RMSEA = .078). Students included in current study (*n* = 158) did not differ significantly from the study sample who participated at baseline only (*n* = 282) on physical activity (69,908 ± 18,786 vs. 67,211 ± 17,655 accelerometer steps/week respectively, paired *t* test, *p* > .05), depressive symptoms (11.62 ± 9.20 vs. 10.30 ± 8.14 respectively, paired *t* test, *p* > .05), and self-esteem (22.02 ± 5.34 vs. 22.26 ± 4.88 respectively, paired *t* test, *p* > .05).

**Table 1:** Descriptive statistics of the study sample

	All (*N* = 158)		Cohort 7^th^ Graders (*N* = 98)		Cohort 9^th^ Graders (*N* = 60)	
	Baseline	Follow-up	Baseline (Grade 7)	Follow-up (Grade 8)	Baseline (Grade 9)	Follow-up (Grade 10)
Age (years)	13.60 ± 1.13	14.62 ± 1.20*	12.79 ± 0.45	13.75 ± 0.42*	14.92 ± 0.47	16.04 ± 0.48*
Sex						
Boys	61 (38.6 %)		38 (38.8 %)		23 (38.3 %)	
Girls	97		60		37	
Nationality						
Native Dutch	140 (88.6 %)		88 (89.8 %)		52 (86.7 %)	
Non-native Dutch	18		10		8	
Educational level						
SGSE	40 (25.3 %)		31 (31.6 %)		9 (15.0 %)	
UPE	118		67		51	
SES						
Low/medium	34 (21.9 %)		21 (21.9 %)		13 (22.0 %)	
High	121		75		46	
Body mass index (kg/m2)	18.75 ± 2.82	19.45 ± 2.83*	17.88 ± 2.81	18.72 ± 2.89*	20.18 ± 2.21	20.65 ± 2.30*
Cardiovascular fitness (VO2max)	51.53 ± 5.51	51.02 ± 6.14*	51.81 ± 5.72	51.70 ± 6.19	51.06 ± 5.17	49.90 ± 5.94*
Total PA (Accelerometer steps)	69908 ± 18786	59229 ± 15021*	73672 ± 18208^B^	58405 ± 13809*^B^	63759 ± 18223^B^	60573 ± 16851^B^
Depressive symptoms	11.62 ± 9.20	10.21 ± 8.95*	10.99 ± 9.01	9.12 ± 8.03* ^B^	12.65 ± 9.49	11.98 ± 10.10 ^B^
Self-esteem	22.02 ± 5.34	22.65 ± 5.30	22.39 ± 5.27	23.49 ± 4.93*^B^	21.42 ± 5.45	21.29 ± 5.62^B^

*SGSE* senior general secondary education, *UPE* university preparatory education, *Total PA* total amount of physical activity per week measured objectively by accelerometer, *VO2max* maximum oxygen consumption

* significantly different from baseline at *p* < .05

^B^ indicates significant difference between 7^th^ graders and 9^th^ graders

### Change in physical activity

The 9^th^ graders (73,672 ± 18,208 accelerometer steps/week) were less active at baseline than the 7^th^ graders (63,759 ± 18,223 accelerometer steps/week, independent *t* test, *p* < .001).

Physical activity levels decreased 15.3 % (10,679 ± 18,965 accelerometer steps/week, paired *t* test, *p* < .001) over the 1-year period. SEM analyses revealed that the change in physical activity was mainly predicted by the baseline level of physical activity (β = -.68, *p* < .001). Higher physical activity levels at baseline predicted sharper decreases after 1-year. Also, the 7^th^ graders (-20.7 %) showed less of a decrease in physical activity after 1-year than the 9^th^ graders (-5.0 %, *p* = .001). The decline in physical activity did not differ significantly between boys (-17.4 %) and girls (-13.8 %, *p* = .910).

### Change in mental health

Overall, depressive symptoms decreased 12.1 % (1.41 ± 7.96) over the 1-year period (paired *t* test, *p* = .027). SEM analyses revealed that the change in depressive symptoms was significantly associated with baseline levels of depressive symptoms (β = -.51, *p* < .001). The higher the baseline levels of depressive symptoms, the greater the decrease over the 1-year period. There was also a significant difference in the change of depressive symptoms in 7^th^ graders (-17.0 %) compared to 9^th^ graders (-5.3 %, *p* = .013). In addition, there was no significant difference in the change of depressive symptoms between boys (-8.1 %) and girls (-14.1 %, *p* = .580).

Overall, self-esteem did not change significantly (2,9 %, 0.64 ± 4.32), over the 1-year period (paired *t* test, *p* = .066). However, there was an interaction between academic year and change in self-esteem. Change in self-esteem was significantly more positive in 7^th^ graders (+4.9 %) than in 9^th^ graders (-0.6 %, *p* = .016). There was no significant difference in the change in self-esteem between boys (+3.0 %) and girls (+2.8 %, *p* = .152).

### Change in physical activity associated with change in mental health over a 1-year period

SEM analyses revealed that changes in physical activity did not seem to predict any changes in depressive symptoms and self-esteem (Fig. 1). The academic year as a control variable was found to be significantly associated with changes in physical activity and self-esteem (see Results above). Baseline levels of cardiovascular fitness appeared to be positively related to physical activity at baseline, but not with the changes in physical activity. Other control variables, including sex, nationality, socioeconomic status, baseline levels of BMI, change in BMI, and change in cardiovascular fitness, were omitted from the analyses because they had no effect on any of the relevant variables.

## Discussion

The main goal of this study was to investigate the associations between changes in physical activity and changes in mental health in adolescents. Over the course of a year, the adolescents’ physical activity levels declined sharply and depressive symptoms decreased; however, no significant overall associations were observed between the changes. This indicates that the decline in physical activity was not associated with a change in the depressive symptoms or self-esteem in adolescents. Changes in depressive symptoms and self-esteem were mainly predicted by their baseline levels. The combination of these findings suggests that the changes in the adolescents’ mental health over the 1-year period are mainly explained by variables other than the decline in physical activity, in particular the baseline measures of mental health.

### Decline in physical activity

In the research reported here, physical activity levels of adolescents declined sharply over a 1-year period. This is in line with the previous studies of Ruiz et al. [15] and Van Mechelen et al. [16] who reported declines in physical activity levels in European and Dutch adolescents respectively. In addition to those studies, our results show that physical activity levels decrease in particular in students in grade 7, the first year of secondary school in the Netherlands. In addition, physical activity decreases more sharply in active students than in inactive students. Increases in sedentary behaviour should be prevented because the physical activity levels developed during adolescence are often maintained during and after the transition to adulthood [39]. In addition, a physically inactive lifestyle during adolescence has been associated with the development of chronic diseases such as cardiovascular diseases and type 2 diabetes later in life [40, 41].

### Change in mental health

Our results show that mental health improved in students progressing from grade 7 to grade 8, but did not change significantly in students progressing from grade 9 to grade 10. The academic year variable might have played an important role in the contradiction between our results and the majority of studies which report increases in depressive symptoms during adolescence [42–44]. In the Netherlands, grade 7 is the first year of secondary school, thus students in grade 7 moved from the seemingly familiar and safe environment of the primary school, to the unfamiliar and strange surroundings of the secondary school. In addition, they often find themselves repositioned as the youngest students in the school and also face the challenge of interacting with new teachers and new peers from different social and cultural backgrounds [45, 46]. Therefore the transition from primary school to secondary school may have resulted in relatively high levels of depressive symptoms in grade 7. Moreover, the findings of this study indicate that when these students progressed to grade 8, they became more familiar with the school, the teachers and their peers, resulting in improved mental health.

A number of studies of adolescents show no change in adolescents’ self-esteem over time [47–49]. Other studies however, reported increases in self-esteem during adolescence [50, 51], which is in line with our results. An explanation for the improvement in self-esteem observed in our study might be that youth with low self-esteem at baseline gain peer approval and avoid rejection by conforming to peer norms and yielding to peer pressure. This may then increase the positive opinions of themselves because they feel validated by their peers [52].

### Decline in physical activity associated with change in mental health

No significant association was found between the decline in physical activity during adolescence and a change in mental health. Our results are in line with findings of Clark et al. [26], Rothon et al. [14] and Lindwall et al. [27], but they also add to these studies by using an objective instrument to determine physical activity. Clark et al. [26] reported that, in 11–14 year old adolescents, baseline levels of physical activity were not significantly associated with depressive symptoms 2 years later. Clark et al. [26] suggested that the relatively short follow-up period of the study might be due to the lack of a significant association, because the pathways between physical activity and mental health may occur later in adolescence or take longer to develop. Our SEM analyses also showed that baseline levels of physical activity did not significantly predict mental health at follow-up (results not published), which might also be due to the relatively short 1-year follow-up period.

Rothon et al. [14] found no significant association between changes in physical activity and changes in depressive symptoms in adolescents progressing from grade 7 to grade 9. Rothon et al. [14] suggested that their self-reported data may partly account for the lack of a significant association between these changes. Our results obtained with the use of accelerometers, support the findings of Rothon et al. [14] (i.e. no association exists between a change in physical activity and a change in mental health) and confirm that this lack of significance cannot be attributed to subjective measurement.

Lindwall et al. [27] reported that a change in physical activity over 3 years was not significantly associated with a change in global self-esteem in 14- and 15-year-old girls. Lindwall et al. [27] suggested that physical activity is more closely linked to physical self-esteem in comparison to global self-esteem, which is situated further away from specific behaviours such as physical activity. This suggestion might also explain the lack of a significant association between the decline in physical activity and changes in self-esteem reported in our study.

Our results are in contrast with the results of Wiles et al. [24] who found that participation in sporting activities (including football and tennis) predicted fewer emotional problems 1-year later. In our study, the total number of accelerometer steps per week was used as the measure of physical activity. This measure gives a reasonable approximation of the total amount of physical activity per week [53], but it does not measure how much time participants spend in (team) sports. Participation in sports and exercise groups might also provide social interaction and promote social support [54], resulting in improved mental health [55]. It might be possible that a combination of physical activity and social interaction, particularly the social support common during sports, compared to physical activity alone, has more beneficial effects on adolescents’ mental health. In conclusion, the addition of a measure of (team) sports participation in our study may have strengthened our research design and perhaps might explain the lack of significant results as well as the difference between our results and the results of Wiles et al. [24].

Our results are in contrast with a number of studies in children and adolescents reporting beneficial effects of physical activity on depressive symptoms (see review of Biddle and Asare [6]). Our main explanation is that many studies in this field included children and adolescents with (clinically) diagnosed depression. Our study sample included only 16 adolescents (10.1 %) who were classified as moderately to severely depressed at baseline, according to the previously mentioned cut off point of Rushton, Forcier and Schectman [43]. It might be possible that physical activity benefits in particular those who experience elevated levels of depressive symptoms, because physical activity leads to an increase in the availability of brain neurotransmitters such as serotonin and dopamine, which are diminished with depression [7]. Of those 16 adolescents who were classified as moderately to severely depressed at baseline, 5 were more active at follow-up than at baseline and 4 of them (80.0 %) were not classified as moderately to severely depressed at follow-up. In addition, the remaining 11 adolescents classified as moderately to severely depressed at baseline were less active at follow-up than at baseline and only 3 of them (27.2 %) were not moderately to severely depressed at follow-up. Therefore, it might be possible that physical activity is particularly beneficial for moderately to severely depressed adolescents. However, additional analyses showed no significant change in depression (i.e. moderately to severely depressed or non-depressed) between baseline and follow-up findings for students who were more or less active).

Finally, it might be possible that physical activity benefits in particular those who experience low levels of self-esteem [56]. In the current study, a total score of self-reported self-esteem was calculated. To sort the participants into a group of high or low self-esteem, a 50 % cut-off point was used in the additional analyses as was done in the study of Boyd and Hrycaiko [28]. A total of 22 adolescents (13.9 %) had low levels of self-esteem based on a cut off score of ≤ 15. Of those, 9 increased their physical activity levels between baseline and follow-up and 5 of them (55.6 %) no longer had low levels of self-esteem at follow-up. In addition, in the 13 adolescents with low levels of self-esteem at baseline who decreased their physical activity levels between baseline and follow-up, 6 (46.1 %) no longer had low levels of self-esteem at follow-up. Additional analyses showed no significant difference in self-esteem (i.e. low versus high levels of self-esteem) for students who increased or decreased their physical activity between baseline and follow-up.

In conclusion, the results of our study reveal no significant association between the decline in physical activity during adolescence and changes in mental health. Our study adds to a limited number of studies in this field, which reported no significant or in most cases weak associations. Therefore, in our opinion, the relationship between changes in physical activity and changes in mental health in adolescents is weak at best.

### Strengths

The major strength of this study is that physical activity was measured objectively at both baseline and follow-up, which has not been the case in previous observational studies on the relationship between physical activity and mental health in adolescents [14, 18, 19, 22–28]. Second, the GOALS study sample included in current study did not differ significantly from the GOALS study sample who participated at baseline only on physical activity, depressive symptoms, and self-esteem. Therefore, the adolescents included in the current study are most likely representative of the association between change in physical activity and change in mental health in our entire GOALS study sample of Dutch adolescents. Third, only complete cases with full data of physical activity and mental health at both time intervals were used for analyses. Fourth, results were controlled for several potential confounders.

### Limitations

The results of this study have to be interpreted with caution due to some limitations. First, the results cannot be generalised to the whole Dutch adolescent population, because the participants were selected from only one secondary school. In addition, only 35.9 % of the entire study sample of The GOALS Study was included in the current study. Second, results were not controlled for (team) sports participation. Third, mental health was measured by self-report instead of by clinical diagnosis. Fourth, both the independent and dependent variables regressed toward the mean. Physical activity levels declined more sharply in active students at baseline than in inactive students at baseline, depressive symptoms declined more sharply in students with high levels of depressive symptoms at baseline than in non-depressed students at baseline and self-esteem improved less in students with high levels of self-esteem at baseline than in students with low levels of self-esteem at baseline. Finally, there is uncertainty about the exact time of the change in physical activity habits. Physical activity levels were measured during a normal school week, which provided a good representation of the adolescents’ physical activity levels. However, the exact point in time that the participants changed their physical activity habits is unknown. Consequently, the duration of time during which changed physical activity habits possibly affected mental health is not controlled for, while this period of time can vary from a matter of days up to one full year. Some beneficial effects of physical activity on mental health act immediately, such as the release of β-endorphins, serotonin or dopamine [7]. Other beneficial effects on mental health may act only when people are physically active for long periods. For example, improvements in perceptions of competence or confidence about the body may be increased through regular physical activity over a long period and this may generalize to mental health [57].

## Conclusions

Our study of Dutch adolescents shows no significant association between the decline in physical activity over a 1-year period and a change in mental health. We observed that changes in mental health were mainly affected by baseline levels of mental health and also the academic year variable seemed to be an important predictor. Future studies of adolescents should make use of rigorous designs to investigate whether a change in physical activity is associated with a change in mental health. We suggest the use of longer follow-up periods (i.e. ≥ 2 years), measurements at multiple time intervals, larger study samples, and control for (team) sports participation, academic year differences and transitions from primary school to secondary school.

### Ethics approval and consent to participate

The Ethical Committee of the Open University of the Netherlands approved the research protocol (reference number: U2013/07405/HVM). Parents and/or guardians were allowed to sign an objection form if they did not agree with the participation of their children, and the students themselves were given the same options. No written consent was obtained as the parents/guardians were informed of the study.

### Consent for publication

This manuscript contains no individual person’s data in any form.

### Availability of data and materials section

The dataset supporting the conclusions of this article is available in the [Publication_Van Dijk et al_PA-Mental Health_BMC Public Health_N = 158] repository in “https://www.dropbox.com/s/k1a3de7k4cl4zpx/160322_Publication%20Van%20Dijk%20et%20al_PA_Mental%20Health_BMC%20Public%20Health_N%3D158.sav?dl=0 [SPSS format].”

## Abbreviations

BMI: body mass index
CES-D: center for epidemiologic studies depression scale
DS: depressive symptoms
PA: physical activity
RSE: Rosenberg self-esteem scale
SE: self-esteem
SGSE: senior general secondary education
UPE: university preparatory education
VO2max: maximum oxygen consumption

## References

[1] Kieling C, Baker-Henningham H, Belfer M, Conti G, Ertem I, Omigbodun O, Rohde LA, Srinath S, Ulkuer N, Rahman A (2011). Child and adolescent mental health worldwide: evidence for action. Lancet.

[2] Weissman MM, Wolk S, Goldstein RB, Moreau D, Adams P, Greenwald S, Klier CM, Ryan ND, Dahl RE, Wickramaratne P (1999). Depressed adolescents grown up. JAMA.

[3] Shaffer D, Gould MS, Fisher P, Trautman P, Moreau D, Kleinman M, Flory M (1996). Psychiatric diagnosis in child and adolescent suicide. Arch Gen Psychiatry.

[4] Egger HL, Costello EJ, Angold A (2003). School refusal and psychiatric disorders: a community study. J Am Acad Child Adolesc Psychiatry.

[5] Paluska SA, Schwenk TL (2000). Physical activity and mental health: current concepts. Sports Med.

[6] Biddle SJ, Asare M (2011). Physical activity and mental health in children and adolescents: a review of reviews. Br J Sport Med.

[7] Craft LL, Perna FM (2004). The benefits of exercise for the clinically depressed. J Clin Psychiatry.

[8] Ekeland E, Heian F, Hagen KB, Abbott J, Nordheim L. Exercise to improve self-esteem in children and young people. Cochrane Database Syst Rev 2004. CD003683. 10.1002/14651858.CD003683.pub2 PMC12935395 14974029

[9] Jackson SA, Marsh HW (1986). Atheletic or antisocial? The female sport experience. J Sport Psych.

[10] Weiss MR, Bredemeier BJ (1990). Developmental sport psychology: a theoretical perspective for studying children in sport. J Sport Exerc Psychol.

[11] Calfas KJ, Taylor WC (1994). Effects of physical activity on psychological variables in adolescents. Ped Exerc Sci.

[12] Ekeland E, Heian F, Hagen KB (2005). Can exercise improve self esteem in children and young people? A systematic review of randomised controlled trials. Brit J Sport Med.

[13] Craft LL, Landers DM (1998). The effect of exercise on clinical depression and depression resulting from mental illness: a meta-analysis. J Sport Psych.

[14] Rothon C, Edwards P, Bhui K, Viner RM, Taylor S, Stansfeld SA (2010). Physical activity and depressive symptoms in adolescents: a prospective study. BMC Med.

[15] Ruiz JR, Ortega FB, Martinez-Gomez D, Labayen I, Moreno LA, De Bourdeaudhuij I, Manios Y, Gonzalez-Gross M, Mauro B, Molnar D, Widhalm K, Marcos A, Beghin L, Castillo MJ, Sjostrom M (2011). Objectively measured physical activity and sedentary time in European adolescents: the HELENA study. Am J Epidemiol.

[16] van Mechelen W, Twisk JW, Post GB, Snel J, Kemper HC (2000). Physical activity of young people: the Amsterdam Longitudinal Growth and Health Study. Med Sci Sports Exerc.

[17] Shephard RJ (2003). Limits to the measurement of habitual physical activity by questionnaires. Br J Sports Med.

[18] Adams SA, Matthews CE, Ebbeling CB, Moore CG, Cunningham JE, Fulton J, Hebert JR (2005). The effect of social desirability and social approval on self-reports of physical activity. Am J Epidemiol.

[19] Duncan GE, Sydeman SJ, Perri MG, Limacher MC, Martin AD (2001). Can sedentary adults accurately recall the intensity of their physical activity?. Prev Med.

[20] Watkinson C, Van Sluijs EMF, Sutton S, Hardeman W, Corder K, Griffin SJ (2010). Overestimation of physical activity level is associated with lower BMI: a Wcross-sectional analysis. Int J Behav Nutr Phys Act.

[21] Warnecke RB, Johnson TL, Chavez N, Sudman S, O’Rourke DP, Lacey L, Horm J (1997). Improving question wording in surveys of culturally diverse populations. Ann Epidemiol.

[22] Motl RW, Birnbaum AS, Kubik MY, Dishman RK (2004). Naturally occurring changes in physical activity are inversely related to depressive symptoms during early adolescence. Psychosom Med.

[23] Neissaar I, Raudsepp L (2011). Changes in physical activity, self-efficacy and depressive symptoms in adolescent girls. Pediatr Exerc Sci.

[24] Wiles NJ, Jones GT, Haase AM, Lawlor DA, Macfarlane GJ, Lewis G (2008). Physical activity and emotional problems amongst adolescents : a longitudinal study. Soc Psychiatry Psychiatr Epidemiol.

[25] Stavrakakis N, De Jonge P, Ormel J, Oldehinkel AJ (2012). Bidirectional prospective associations between physical activity and depressive symptoms. The TRAILS study. J Adolesc Health.

[26] Clark C, Haines MM, Head J, Klineberg E, Arephin M, Viner R, Taylor SJ, Booy R, Bhui K, Stansfeld SA (2006). Psychological symptoms and physical health and health behaviours in adolescents: a prospective 2-year study in East London. Addiction.

[27] Lindwall M, Asci H, Crocker P (2014). The physical self in motion: within-person change and associations of change in self-esteem, physical self-concept, and physical activity in adolescent girls. J Sport Exerc Psychol.

[28] Boyd KR, Hrycaiko DW (1997). The effect of a physical activity intervention package on the self-esteem of pre-adolescent and adolescent females. Adolescence.

[29] Tremblay MS, Inman JW, Willms JD (2000). The relationship between physical activity, self-esteem, and academic achievement in 12-year-old children. Ped Exerc Sci.

[30] Schmalz DL, Deane GD, Birch LL, Krahnstoever Davison L (2007). A longitudinal assessment of the links between physical activity and self-esteem in early adolescent non-hispanic females. J Adolesc Health.

[31] Van Dijk ML, De Groot RHM, Savelberg HCM, Van Acker F, Kirschner PA (2014). The association between objectively measured physical activity and academic achievement in Dutch adolescents: findings from the GOALS study. J Sport Exerc Psychol.

[32] Trost SG, McIver KL, Pate RR (2005). Conducting accelerometer-based activity assessments in field-based research. Med Sci Sport Exerc.

[33] Radloff LS (1977). The CES-D scale: a self-report depression scale for research in the general population. App Psychol Measure.

[34] Rosenberg M (1979). Conceiving the self.

[35] Leger LA, Mercier D, Gadoury C, Lambert J (1988). The multistage 20 metre shuttle run test for aerobic fitness. J Sports Sci.

[36] R Core Team 2013. R: A language and environment for statistical computing. R Foundation for Statistical Computing, Vienna, Austria. Avaiable from: http://www.R-project.org. Accessed 5 Mar 2013.

[37] Rosseel Y (2012). lavaan: An R package for structural equation modeling. J Stat Software.

[38] Dall PM, McCrorie PR, Granat MH, Stansfield BW (2013). Step accumulation per minute epoch is not the same as cadence for free-living adults. Med Sci Sport Exerc.

[39] Hallal PC, Victora CG, Azevedo MR, Wells JC (2006). Adolescent physical activity and health: a systematic review. Sports Med.

[40] Ortega FB, Konstabel K, Pasquali E, Ruiz JR, Hurtig-Wennlof A, Maestu J, Lof M, Harro J, Bellocco R, Labayen I, Veidebaum T, Sjostrom M (2013). Objectively measured physical activity and sedentary time during childhood, adolescence and young adulthood: a cohort study. PLoS One.

[41] Caspersen CJ, Pereira MA, Curran KM (2000). Changes in physical activity patterns in the United States, by sex and cross-sectional age. Med Sci Sport Exerc.

[42] Kaplan SL, Hong GK, Weinhold C (1984). Epidemiology of depressive symptomatology in adolescents. J Am Acad Child Adolesc Psychiatry.

[43] Rushton JL, Forcier M, Schectman RM (2002). Epidemiology of depressive symptoms in the National Longitudinal Study of Adolescent Health. J Am Acad Child Adolesc Psychiatry.

[44] Saluja G, Iachan R, Scheidt PC, Overpeck MD, Sun W, Giedd JN (2004). Prevalence of and risk factors for depressive symptoms among young adolescents. Arch Ped Adolesc Med.

[45] Pratt S, George R (2006). Transferring friendship: girls’ and boys’ friendships in the transition from primary to secondary school. Child Soc.

[46] West P, Sweeting H, Young R (2010). Transition matters: pupils’ experiences of the primary–secondary school transition in the West of Scotland and consequences for well‐being and attainment. Res Pap Educ.

[47] Brack CJ, Orr DP, Ingersoll G (1988). Pubertal maturation and adolescent self-esteem. J Adolesc Health Care.

[48] Chubb NH, Fertman CI, Ross JL (1997). Adolescent self-esteem and locus of control: a longitudinal study of gender and age differences. Adolescence.

[49] Savin-Williams RC, Demo DH (1984). Developmental change and stability in adolescent selfconcept. Dev Psychol.

[50] McCarthy JD, Hoge DR (1982). Analysis of Age Effects in Longitudinal Studies of Adolescent Self-Esteem. Dev Psych.

[51] Bachman JG, O’Malley PM (1977). Self-esteem in young men: a longitudinal analysis of the impact of educational and occupational attainment. J Pers Soc Psychol.

[52] Zimmerman MA, Copeland LA, Shope JT, Dielman TE (1997). A longitudinal study of self-esteem: implications for adolescent development. Am Educ Res J.

[53] Tudor-Locke C, Craig CL, Beets MW, Belton S, Cardon GM, Duncan S, Hatano Y, Lubans DR, Olds TS, Raustorp A, Rowe DA, Spence JC, Tanaka S, Blair SN (2011). How many steps/day are enough? for children and adolescents. Int J Behav Nutr Phys Act.

[54] Sagatun A, Sogaard AJ, Bjertness E, Selmer R, Heyerdahl S (2007). The association between weekly hours of physical activity and mental health: a three-year follow-up study of 15-16-year-old students in the city of Oslo, Norway. BMC Pub Health.

[55] Eime RM, Young JA, Harvey JT, Charity MJ, Payne WR (2013). A systematic review of the psychological and social benefits of participation in sport for children and adolescents: informing development of a conceptual model of health through sport. Int J Behav Nutr Phys.

[56] Barnett NP, Smoll FL, Smith RE (1992). Effects of enhancing coach-athlete relationships on youth sport attrition. Sport Psychol.

[57] Fox KR (1999). The influence of physical activity on mental well-being. Public Health Nutr.

